# White Laue and powder diffraction studies to reveal mechanisms of HCP-to-BCC phase transformation in single crystals of Mg under high pressure

**DOI:** 10.1038/s41598-023-29424-z

**Published:** 2023-02-07

**Authors:** Evgenii Vasilev, Dmitry Popov, Maddury Somayazulu, Nenad Velisavljevic, Marko Knezevic

**Affiliations:** 1 Depatment of Mechanical Engineering, University of New Hampshire, Durham, NH 03824 USA; 2 X-Ray Science Division, High Pressure Collaborative Access Team, Argonne National Laboratory, Argonne, IL 60439 USA; 3 Physics Division, Lawrence Livermore National Laboratory, Livermore, CA 94550 USA

**Keywords:** Engineering, Materials science

## Abstract

Mechanisms of hexagonal close-packed (HCP) to body-centered cubic (BCC) phase transformation in Mg single crystals are observed using a combination of polychromatic beam Laue diffraction and monochromatic beam powder diffraction techniques under quasi-hydrostatic pressures of up to 58 ± 2 GPa at ambient temperature. Although experiments were performed with both He and Ne pressure media, crystals inevitably undergo plastic deformation upon loading to 40–44 GPa. The plasticity is accommodated by dislocation glide causing local misorientations of up to 1°–2°. The selected crystals are tracked by mapping Laue diffraction spots up to the onset of the HCP to BCC transformation, which is determined to be at a pressure of 56.6 ± 2 GPa. Intensity of the Laue reflections from HCP crystals rapidly decrease but no reflections from crystalline BCC phase are observed with a further increase of pressure. Nevertheless, the powder diffraction shows the formation of 110 BCC peak at 56.6 GPa. The peak intensity increases at 59.7 GPa. Upon the full transformation, a powder-like BCC aggregate is formed revealing the destructive nature of the HCP to BCC transformation in single crystals of Mg.

Magnesium (Mg) and its alloys have been studied for decades with more and more interest attracted in recent years. In engineering, Mg alloys are prospective due to their lightweight and high specific strength which is especially important in electronic and transportation industries^1–3^. In geological sciences, Mg is a component of commercially important minerals such as dolomite, magnesite, brucite, carnallite, and olivine. As formation of these minerals takes place at high pressures, it is of fundamental interest to understand phase transformations occurring in the material subjected to these conditions.

In 1985, Olijnyk and Holzapfel^4^ experimentally observed BCC-HCP transformation in Mg in the range of 50 ± 6 GPa and in 2014, Stinton et al.^5^ confirmed this range. However, both works used nonhydrostatic loading conditions leading to uncertainties of the nominal pressure. In^4^, isopropanol was used as pressure-transmitting medium (PTM). In alcohols used as PTM, pressure deviation can reach as high as 2.5 GPa at 20 GPa of average pressure^6^. In^5^, no pressure-transmitting medium was used at all. On top of that, powder samples and powder diffraction measurements used in^4,5^ provided no information on mechanism of the transformation. Further theoretical studies estimated transformation pressure as high as 65 GPa^7–10^. With such broad estimates, it is crucial to not only experimentally verify HCP to BCC transformation pressure but also reveal its mechanism for large crystalline aggregates such as mono- or polycrystals.

In this work, we combined polychromatic beam Laue diffraction and monochromatic powder diffraction at the Advanced Photon Source, High Pressure Collaborative Access Team (HPCAT) to study mechanisms of HCP-BCC phase transformation in Mg single crystals at ambient temperature and quasi-hydrostatic pressure up to 58 ± 2 GPa^11,12^.

Specimens of pure Mg with nominal purity 99.9 + % were cut from a bulk Mg single crystal into small pieces with a thickness of ~ 10 um using a laser drilling machine at HPCAT^13^. Each sample was put into a diamond anvil cell (DAC) BX90 using a micromanipulator^12^. All samples were loaded in DAC in a way that basal plane of HCP crystal is perpendicular to the X-ray beam and parallel to the DAC plane. For detailed description and illustration of DAC reader is referred to^14^. DACs with culets 300 and 200 μm in diameter were used for different samples. The smaller culet allows achieving higher pressure but causes higher stress gradient^15–17^. Rhenium (Re) gasket was pre-indented down to 35 μm and a hole of 150 or 100 μm diameter was drilled in the gasket for 300 and 200 um culets respectively. After loading to the highest pressure, samples were investigated by optical or electron microscopy to determine presence of bridging (squishing) samples between diamonds. No bridging was observed after loading to pressures as high as 58 ± 2 GPa except of partial bridging of one sample illustrated in the supplementary material of the paper. Seven samples were tested with 300 μm culets anvils and three samples were tested with 200 μm culet anvils. Among them, only four samples loaded with culets 300 μm and Neon (Ne) as the transmitting medium were successfully tested at pressures beyond 40 GPa. Other samples exhibited significant plastic deformation to the extent of complete disappearance of diffraction spots as described below. The difference between culet size 300 μm versus 200 μm likely comes from different pressure gradient across the culet. It is known that smaller culets produce a larger pressure gradient^18,19^ which would result in larger plastic deformation of Mg crystals.

The DACs with samples were loaded with Ne or Helium (He) as a pressure transmitting medium. Pressure in the cell was manually increased by set screws in 3–4 GPa increments. Applied pressure was measured by ruby fluorescence^20^ and/or Raman spectrometry on diamond, in order to avoid squeezing of the samples between gasket material and Ruby balls^21^. Difference of measured pressure by these two techniques was no more than 1 GPa. As DACs typically have some pressure drifts even without an increase of the pressure, the estimated drift ranged ± 2 GPa from the initially measured value. Data acquisition was performed at stations 16-BMB^12^ and 16-BMD^22^ for polychromatic Laue and monochromatic powder diffraction techniques respectively. Diffraction patterns were collected over a 2D array of points to cover the entire projection of the sample perpendicular to the beam. These steps were repeated as the sample was compressed up to the highest pressure. The sample was periodically re-centered on the rotation axis by doing absorption scans with a photodiode to keep the sample at the same position with respect to the X-ray beam and area detector^17,18^.

The data analysis procedure was performed using in-house developed MATLAB-based software *IndexLaue*. The software combines functions of image processing in Dioptas^25^, peak search in Fit2d^26^, and indexing in polyLaue^12^ with accelerated multi-core performance and convenient graphical user interface. Full details regarding the functionality of the software are beyond the scope of this publication.

Typical workflow of data processing in IndexLaue is illustrated in Fig. 1. A raw detector image (Fig. 1a) is first enhanced by increasing brightness and contrast (Fig. 1b). Then, reflections from diamond are subtracted and a reflection of interest is selected for mapping (Fig. 1c, where red square indicates the selected reflection). Then, detector images are cropped to the selection box and plotted according to X and Y axes of the scan. As a results, a 2D map of the selected reflection (area) is plotted following the scan dimensions (Fig. 1d). Resultant map shows a spatial location of a crystal that produces the selected diffraction spot. Finally, reflections are indexed to identify the phase and orientation of the diffracting crystal and Miller Indices (hkl) of the diffraction spots. Figure 1e, f shows an example of such indexing in which diffraction spots are found to belong to a single crystal of HCP Mg.

**Figure 1:**
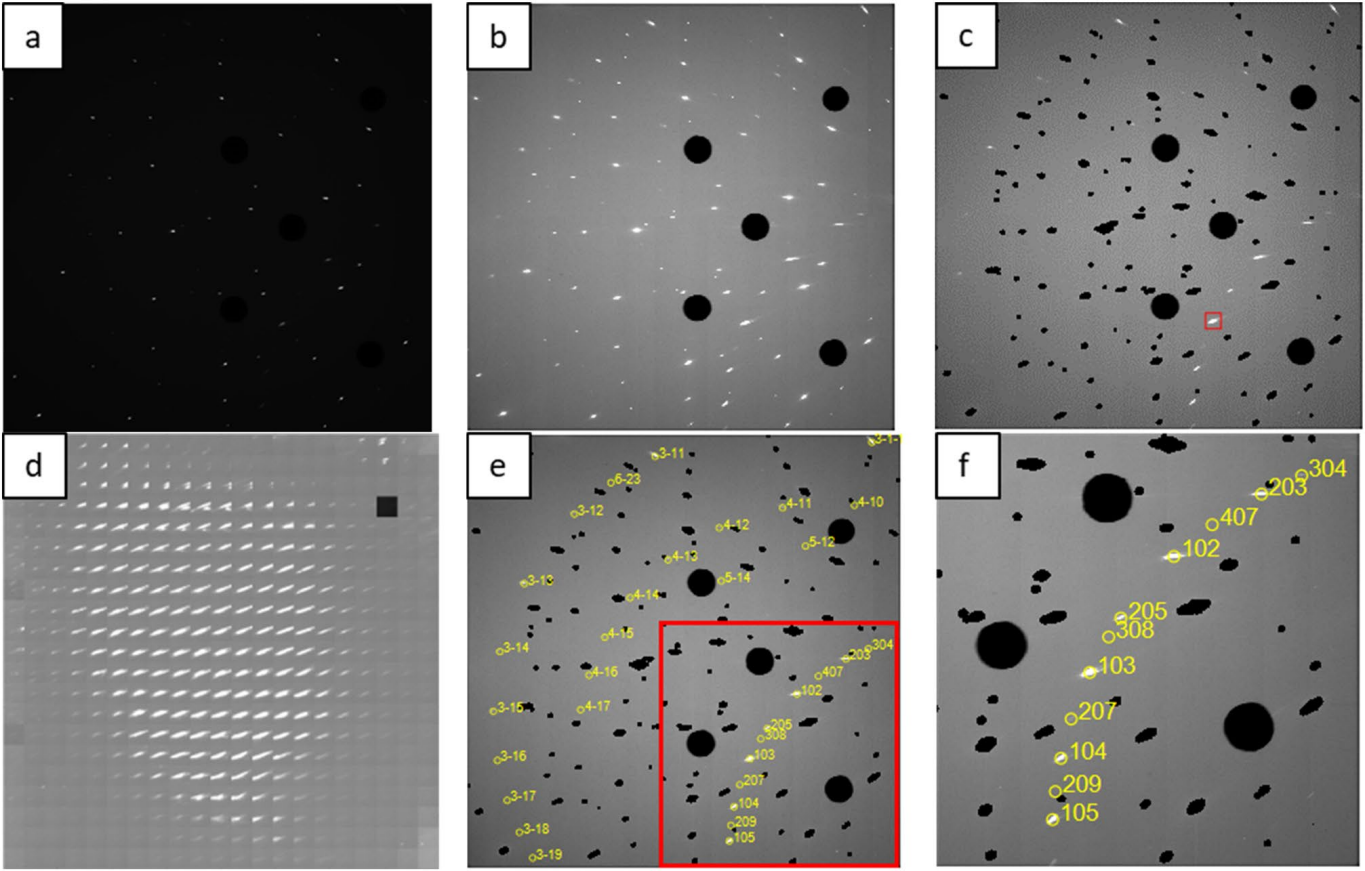
(**a**) Initial detector image, (**b**) detector image with adjusted brightness and contrast, (**c**) adjusted detector image with masked diamonds; red square indicates the diffraction spot used for mapping, (**d**) 2D map of the diffraction spot selected in (**c**), (**e**) indexed diffraction spots with Miller Indices (hkl) labels; red square indicates magnified portion presented in (**f**).

Initial pressure on the sample is slowly increased up to 40…44 GPa, which is close to the expected lower bound of the HCP-BCC transition 44 GPa^4^. As the pressure was increased, samples exhibited some plastic deformation, even before any phase transformation could potentially occur (Fig. 2, supplementary material of the paper). Diffraction spots that are reasonably sharp at 5 GPa (Fig. 2a,d) and 22 GPa (Fig. 2b,e), become diffuse when reaching 41.2 GPa (Fig. 2c,f). The effect was much more pronounced for DACs having culet size of 200 um, to the extent that no indexable diffraction spots were observed at 40 GPa on three samples tested with this culet size. Thus, we used 300 um culets to minimize deformation of crystals in the rest of the experiments.

**Figure 2:**
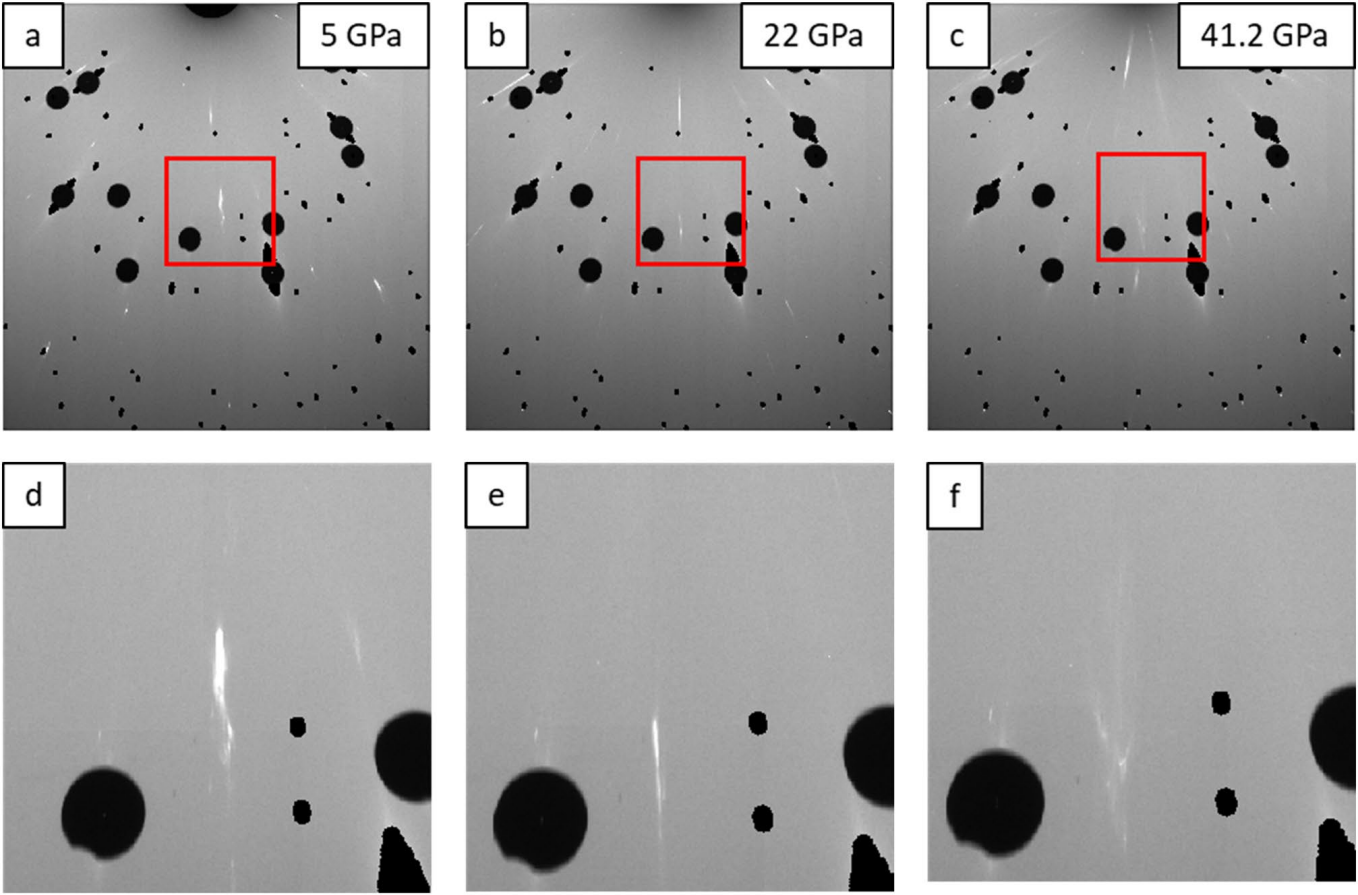
Series of images illustrating deformation of Mg single crystal with increasing of pressure in DAC: (**a**)–(**c**) Detector images at 5, 22, and 41.2 GPa respectively; red squares represent magnified areas shown on (**d**)–(**f**). Note decrease of intensity and diffuse appearance of the reflections in (**c**) and (**f**). Pressure medium was He.

The deformation of Mg crystals likely happens due to non-hydrostatic component of the applied pressure which results in dislocation slip and formation of local misorientations up to 1°-2° in the probed volume. Such misorientation corresponds to 100–200 pixels spread on the detector images forming a diffuse cloud instead of a sharp diffraction spot. No difference was observed between He (Fig. 2) and Ne (supplementary material of the paper) pressure mediums as plastic deformation of Mg happened in both to a similar extent. Though He is known to be the most hydrostatic pressure medium within the studied pressure range , the value of non-hydrostatic effect (pressure deviation) in He rises starting from ~ 22.5 GPa^6^. At pressure 40 GPa, the pressure deviation in He is estimated to be 0.15 GPa = 150 MPa. Though experimental conditions vary between our test and^6^, it is not surprising that crystals of pure Mg would deform at non-hydrostatic pressures exceeding flow stress of Mg which can be as low as few MPa^27^. As basal slip is the easiest in Mg^27–30^, we can expect early activation of this slip mode given that Schmidt factor is not equal to zero. However, non-basal slip can also be activated with an increase of non-hydrostatic component of applied pressure and in favorable orientation of the crystal^31,32^. While active slip modes and change of dislocation density can be measured by monochromatic diffraction methods^33,34^, this lies beyond the scope of this work. We thus estimate only the slip-induced local misorientation that can be readily inferred from size of Laue reflections. When it comes to twinning as mechanisms of accommodating plasticity in Mg^35–38^, no twins of detectable size were observed at any stage of loading up to the highest pressure.

Thus, present observations clearly confirm the non-hydrostatic effect of He and Ne pressure mediums. This should be accounted in future studies on Mg crystals subjected to high pressure, as deformation is inevitable in the range beyond ~ 20 GPa leading to smearing of diffraction spots. In attempt to reduce plastic deformation, annealing at 375 °C for 6 h was performed on one of the samples pre-loaded to 42 GPa, however, no improvement of diffraction spots was observed.

We then continue with loading samples through the transformation range, previously established in the literature as 44–56 GPa^4^. Four samples loaded with Ne were successfully tested and showed similarity of observations, which are as follows. Figure 3 shows evolution of detector images (Fig. 3a,c,e,g) and corresponding 2D maps (Fig. 3b,d,f,h) across the pressure range from 42 to 58 GPa. 2D maps were created from the diffraction spot, indicated by a red box on Fig. 3b,d,f,h. This diffraction spot was selected from the same crystal of HCP Mg at all pressures, thus, 2D maps represent spatial location of this crystal, highlighted by orange dash oval outline, and its evolution across the transformation range.

**Figure 3:**
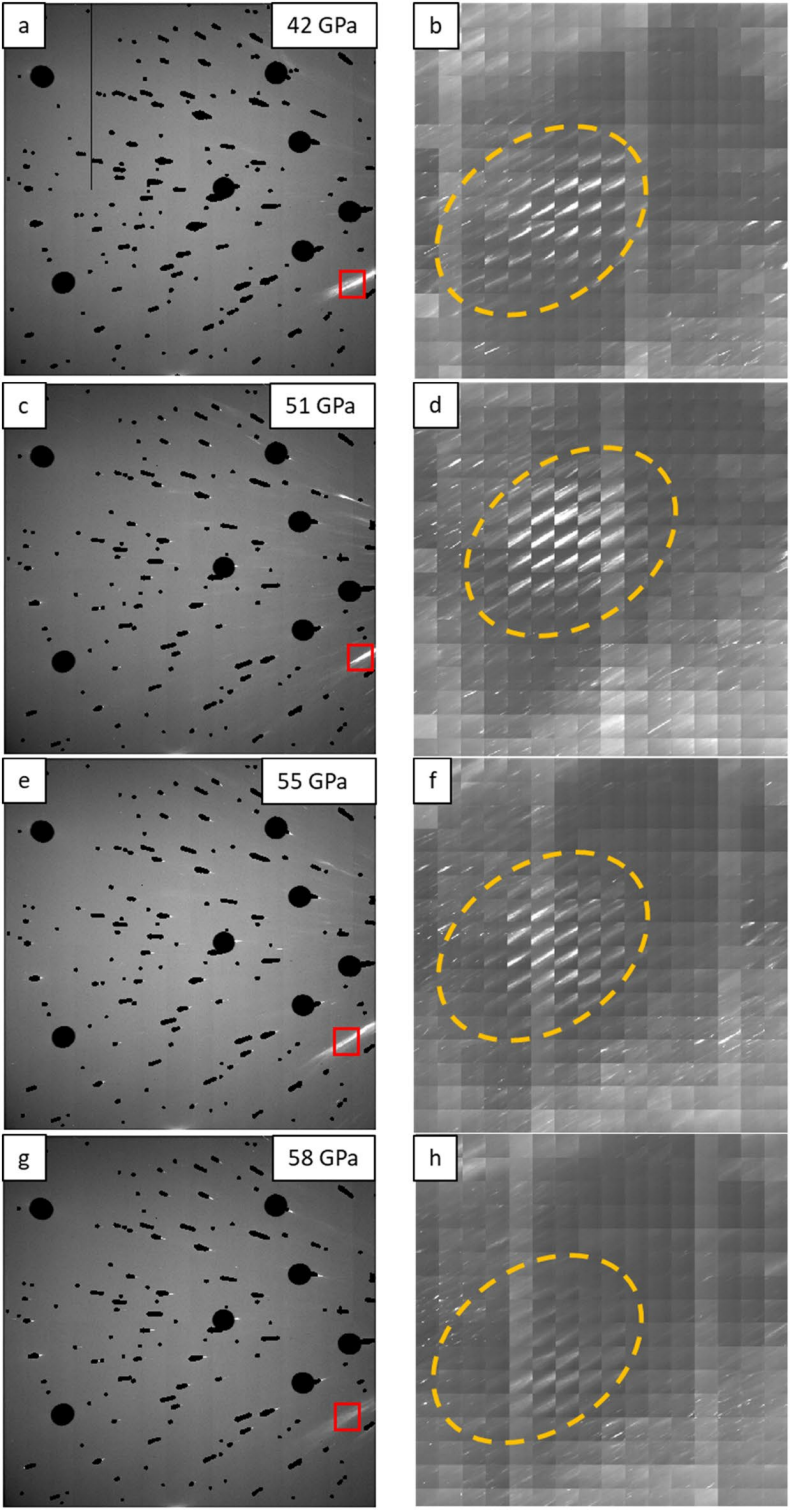
Series of images illustrating evolution of a Mg single crystal through the pressure range 42–58 GPa: (**a**), (**c**), (**e**), (**g**) Detector images; red boxes indicate the diffraction spot used to create 2D maps presented on (**b**), (**d**), (**f**), (**h**) respectively. Orange dash ovals on (**b**), (**d**), (**f**), (**h**) outline a spatial location of a Mg single crystal. Note significant drop of the intensity from the reflection of interest upon pressure increase from 55 GPa (**f**) to 58 GPa (**h**). Pressure medium was Ne.

As we can see from the maps presented on Fig. 3b,d,f, orange dash ovals outline very similar shape of the selected Mg crystal that remains almost intact in the pressure range 42–55 GPa. We then note significant drop of the intensity from the selected reflection upon pressure increase from 55 GPa (Fig. 3f) to 58 GPa (Fig. 3h) while no new reflections appear. Such drop of intensity could indicate occurring phase transformation from HCP to BCC, though no diffraction spots from BCC phase were reliably identified on the Laue diffraction patterns.

Collection of the polychromatic Laue diffraction data was followed by monochromatic powder diffraction measurements to further resolve the BCC crystals. Data collection was performed on a separate sample as it was not possible to combine Laue and Powder diffraction on a single sample due to availability of the beamtime. Resulting spectra are presented on Fig. 4 for pressure range from 41.2 to 59.7 GPa. At the lowest pressure, 5 diffraction peaks were identified in the displayed d_hkl_ range, namely 100 and 101 from Mg HCP, 100 and 101 from Rhenium (Re) gasket, and one diffraction line from solidified crystalline Ne. Upon increasing pressure, 100 and 101 peaks from Mg HCP and Re merged together, and at 56.6 GPa, a minor peak from Mg BCC is observed which then significantly increased in intensity at 59.7 GPa. The appearance of diffraction ring from BCC Mg first resembled a textured aggregate at 56.6 GPa which transformed at 59.7 GPa into nearly-continuous 110 ring typical for powder-like nano-crystalline aggregates, with mostly arbitrary crystal orientations. Atomic volumes derived from positions of diffraction lines of Mg HCP and BCC (Table 1) are in good agreement with the previously reported values^5^.

**Figure 4:**
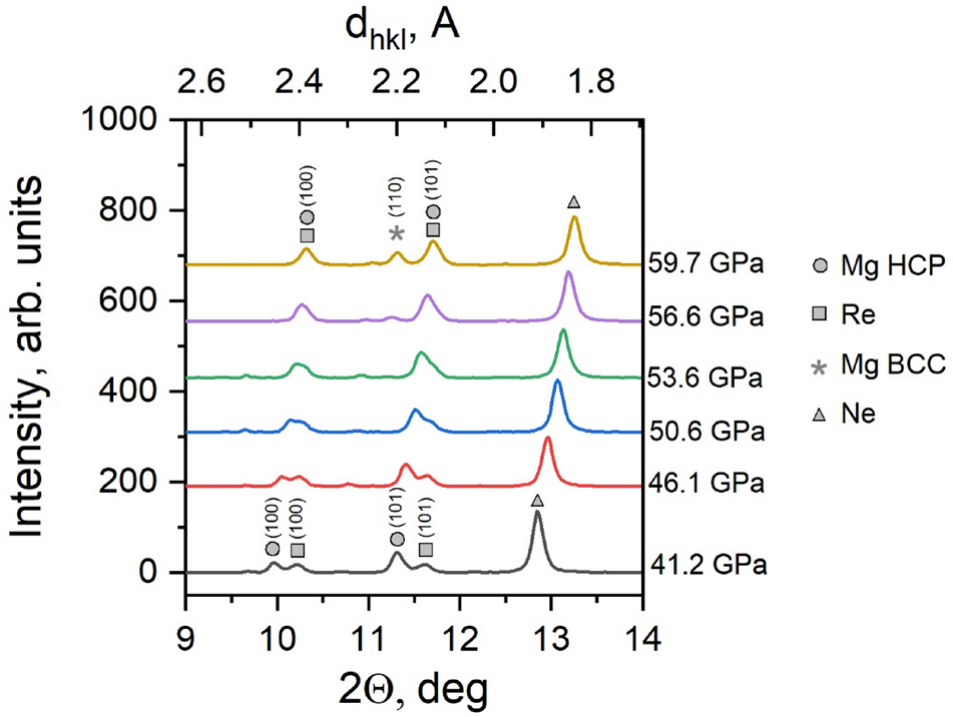
Powder diffraction spectra from Mg single crystal subjected to pressures in the range from 41.2 to 59.7 GPa.

**Table 1:** Atomic volumes derived from positions of diffraction lines of Mg HCP and BCC.

Pressure, GPa	HCP			BCC	
	d(100), Å	d(101), Å	Atomic volume, Å^3^	d(110), Å	Atomic volume, Å^3^
41.2	2.380	2.096	14.5		
46.1	2.359	2.081	14.2		
50.6	2.337	2.059	13.7		
53.6	2.321	2.048	13.5		
56.6				2.109	13.3
59.7				2.096	13.0

From powder diffraction data we can conclude that the lower bound of transformation pressure for pure single crystalline Mg was found to be 56.6 ± 2 GPa with confirmed presence of BCC phase. The obtained value is higher compared to previously established 49 GPa on powder samples^4^. This difference can be explained by different microstructure of the samples, namely single crystal(s) in the present study and powder in^4^. Powder sample essentially represents an aggregate of randomly oriented crystals; even though each single crystal is anisotropic, the average behavior of the aggregate is isotropic. This is especially important for HCP lattice which is highly anisotropic with respect to *c* and *a* axes.

In studies of plasticity under non-hydrostatic loading conditions, orientation of the crystal in respect to the loading direction is critical to understand the loading response. In high-pressure studies, if pressure medium is ideal and does not have non-hydrostatic component, the crystal is compressed completely hydrostatically, and orientation of the crystal in DAC is not important. In the case of real pressure medium, there is always a pressure deviation across DAC, and this pressure deviation will lead to plastic deformation of the sample if flow stress is exceeded. Intuitively, this plastic deformation would be different for different orientations of the sample in DAC. Subsequently this would produce a different microstructure, for example, different dislocation density, in the sample before approaching the phase transformation pressure. Finally, as it was mentioned above, different microstructures might result in different transformation pressure.

As all samples in the present study were cut and loaded in DAC with similar orientation, in the experiment we measure transformation pressure resulted from the microstructure produced by deformation of this particular orientation of the HCP lattice. In contrast, in powder samples we measure the “averaged” properties of the entire aggregate with random orientation of crystallites. In addition to that, grain/phase boundaries in compacted powder or polycrystal present defects with stored energy that can act as nucleation sites and lower the energy barrier required for changes of the state such as phase transformation.

From Laue diffraction data, it can be seen that upon loading, the crystals of HCP Mg exhibited cold working in both Ne and He pressure mediums, though large crystals retain similar shape outline till the onset of phase transformation. In the pressure range of 55–58 GPa, intensity of Laue reflections from HCP crystals significantly dropped while no new Laue reflections were observed from BCC phase. This was accompanied by the formation of 110 BCC powder diffraction peak at 56.6 GPa, which significantly increased in intensity at 59.7 GPa indicating the ongoing HCP-BCC phase transition.

Lack of new Laue reflections from BCC phase combined with the formation of 110 BCC peak on powder diffraction in the range of 55–59.7 GPa indicated that HCP-BCC transformation in Mg is destructive to the crystalline state of the HCP phase with formation of powder-like BCC phase. This conclusion is fundamentally important for understanding the nature of the phase transformation in Mg, as no similar mechanism was described in previous studies that used powder samples and sole powder diffraction techniques. It should be noted that we use a term “powder-like BCC phase” not in the meaning or loose powder, such as grains of sand (which would be highly compacted under high pressure), but rather the same bulk sample whose microstructure transformed from a single HCP crystal into many BCC crystallites with random orientation. Though these crystallites are still kept together in a bulk solid of the initial sample, they are very small and randomly oriented to produce continues diffraction rings similar to ones obtained from loose powder. The statement that sample remains bulk is supported in supplementary material of the paper with an illustration of the sample unloaded to the atmospheric pressure. If loose powder is formed upon transformation, it would remain powder upon unloading; in contrast, we observe that sample remains bulk solid after unloading.

When it comes to the reverse transformation from BCC to HCP upon unloading, we did not specifically address this in the present work as it was previously studied in^4^. A large hysteresis of transformation pressure was observed, reaching 56 GPa for BCC to HCP transformation and 44 GPa for the reverse. That is why authors referred the transformation pressure to a range of 50 ± 6 GPa. In the future, similar unloading experiments can be performed on single crystals along with study of transformation pressure on different orientations of the single crystal.

We should also note that effect of grain fragmentation can be studied using the Scherrer’s equation^39^ by determination of peak broadening in powder diffraction on powder samples, however there are certain complications of its application, namely: (1) Scherrer’s equation is primarily applicable to crystallite sizes less than 10 nm, and if newly formed crystallites are larger than this, there will be no significant peak broadening, (2) peak broadening is comprised of broadening due to the crystallite size and broadening due to imposed strain, while the latter one can be difficult to determine in this type of experiments, (3) the degree of refinement and associated peak broadening will depend on the initial particle size; if initial crystals are fine enough, further refinement might be suppressed.

In contrast, single crystal samples provide distinct diffraction spots that are easier to track down and analyze, particularly by white beam Laue diffraction technique that is much more sensitive to single crystals than powder diffraction. If a deformed single crystal is subjected to powder diffraction, diffraction spots will appear as arcs on Debye-Sherrer ring and likely overlap with any other crystals of the same phase. In contrast, on white Laue diffraction pattern, these crystals can be separated relatively easily. Thus, a combination of polychromatic Laue and monochromatic powder diffraction techniques in this work utilizes strengths of each technique and provides unique insight into the studied phenomena. While powder diffraction was more suitable for determination of phase composition, Laue diffraction gave insights into the crystalline state of the material.

Besides revealing the destructive nature of HCP-BCC transformation, white Laue diffraction highlights important technological aspects of synchrotron diffraction study of Mg at high pressure. This includes pronounced cold deformation of crystals below the transformation range, and non-hydrostatic component of He pressure medium at pressures below the onset of phase transformation. Both of these observations should be considered for further studies on Mg subjected to high pressure.

Application of Laue diffraction to samples which exhibit such strong plastic deformation makes it possible to recognize single crystals much more efficiently than using monochromatic beam. Laue diffraction technique is not sensitive to powder-like nano-crystalline aggregates which either produce very diffuse and “streaky” reflections or just give rise to background while single-crystals produce sharp reflections. If monochromatic beam is used, continuous diffraction lines from powder-like components in general overlap with sharp reflections produced by single-crystals. By application of Laue diffraction, it was demonstrated that, despite of severe plastic deformation, portions of Mg single crystals retain even at pressures right below the HCP to BCC transition, probably due to substantially lower density of dislocations before compression. This information may be of interest from technological point of view, particularly to study of soft materials prone to deformation due to non-hydrostatic pressure conditions.

The problem of diffuse reflections from deformed materials can be reduced to a certain extent with smaller x-ray beam size. Smaller probed volume would result in lesser orientation spread and thus sharper reflections that are easier to track and index. This will improve diffraction pattern from crystalline materials exhibiting plastic deformation as plasticity is shown to be inevitable even when using the most hydrostatic pressure mediums.

Though reduction of beam size can be done through focusing optics and increasing the focusing distance, the largest benefits will be achieved after an upgrade of the entire synchrotron facility to the next generation. For example, after the upcoming APS-U upgrade^40^, beam size is expected to be reduced from 2 um down to ~ 200 nm which will highly benefit studies similar to the present one. In addition to general improvement of the beam brightness and divergence after the upgrade, upcoming combination of monochromatic powder diffraction and polychromatic white Laue diffraction techniques at one station of sector 16 will bring much broader capabilities to material analysis under high pressure.

## Supplementary Information

Supplementary Figures.

## Data Availability

The datasets used and/or analyzed during the current study available from the corresponding author on reasonable request.
