# Supplementary material for: White Laue and powder diffraction studies to reveal mechanisms of HCP-to-BCC phase transformation in single crystals of Mg under high pressure
Source: Sci Rep. 2023 Feb 7;13:2173. doi: 10.1038/s41598-023-29424-z (PMC9905478; doi:10.1038/s41598-023-29424-z)
Supplement: Supplementary file 1 — Supplementary Figures. [file 41598_2023_29424_MOESM1_ESM.docx]

This supplementary material provides additional illustrations of partial bridging (Figure A1) and deformation (Figure A2).


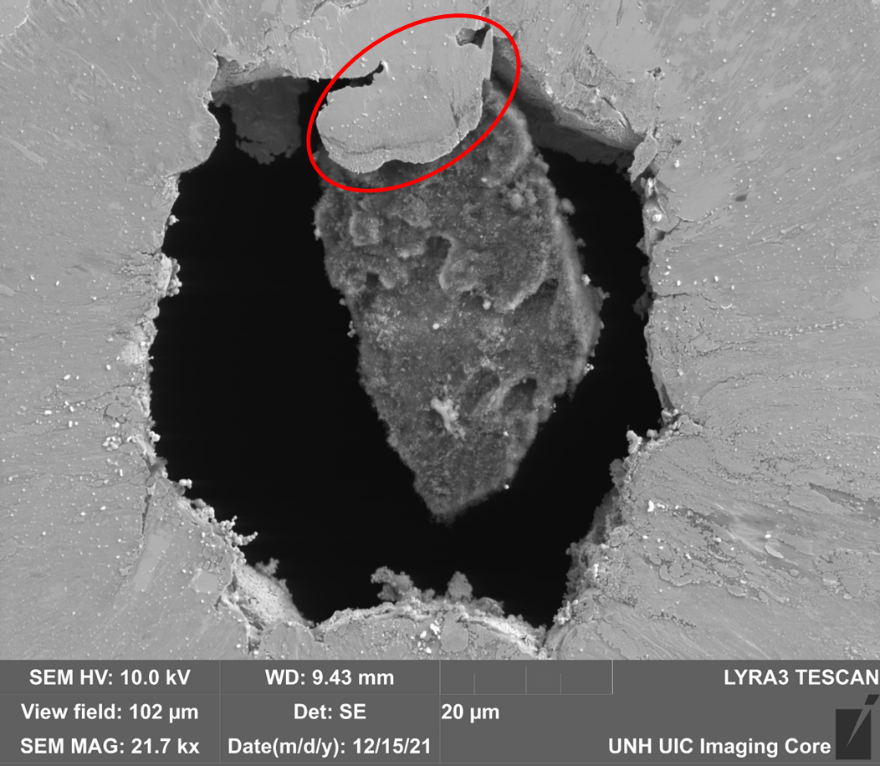


Figure A1 – Scanning Electron Microscope (SEM) image illustrating partial bridging (squishing) of the sample in the area highlighted by the red circle. Maximum pressure achieved in the sample is 52.0±2 GPa.

| 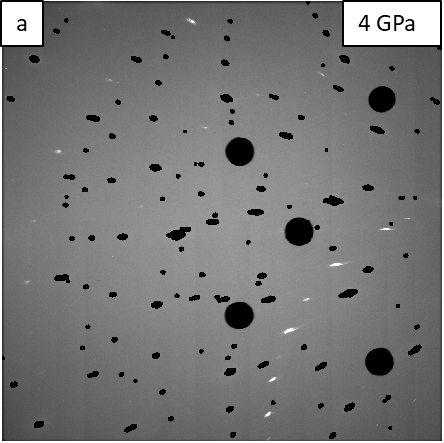 | 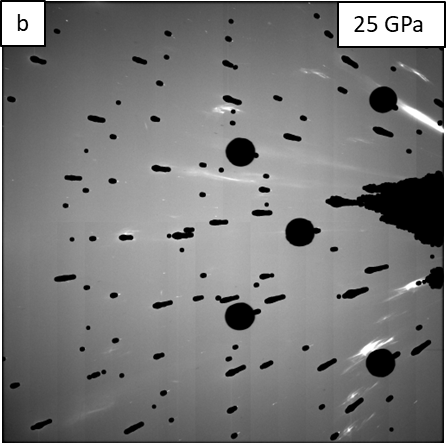 | 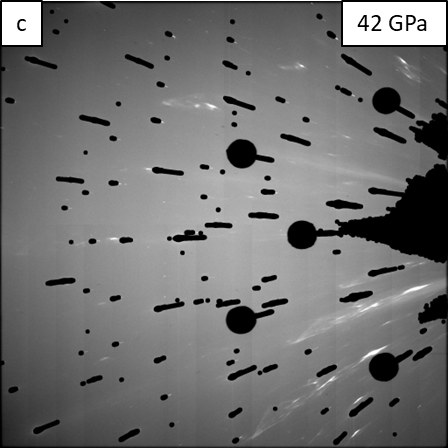 |
| --- | --- | --- |

Figure A2 – Series of images illustrating deformation of Mg single crystal with increasing of pressure in DAC: Detector images at 4, 25, and 42 GPa respectively. Note diffuse appearance of reflections on (b) and (c). Pressure medium was Ne.
